# *In vitro* Activation of heme oxygenase-2 by menadione and its analogs

**DOI:** 10.1186/2045-9912-4-4

**Published:** 2014-02-18

**Authors:** Dragic Vukomanovic, Mona N Rahman, Yaroslav Bilokin, Andriy G Golub, James F Brien, Walter A Szarek, Zongchao Jia, Kanji Nakatsu

**Affiliations:** 1 Department of Biomedical & Molecular Sciences, School of Medicine, Queen’s University, Kingston, ON K7L 3 N6, Canada; 2 Department of Chemistry, Queen’s University, Kingston, ON K7L3N6, Canada; 3 Otava Ltd., Toronto, Ontario M2N 1Y1, Canada; 4 Department of Medicinal Chemistry, Institute of Molecular Biology and Genetics of the National Academy of Sciences of Ukraine, 150 Zabolotnogo Street, Kyiv 03680, Ukraine

**Keywords:** Heme oxygenase, Enzyme activator, Menadione, Agonist, In vitro, Redox properties

## Abstract

**Background:**

Previously, we reported that menadione activated rat, native heme oxygenase-2 (HO-2) and human recombinant heme oxygenase-2 selectively; it did not activate spleen, microsomal heme oxygenase-1. The purpose of this study was to explore some structure–activity relationships of this activation and the idea that redox properties may be an important aspect of menadione efficacy.

**Methods:**

Heme oxygenase activity was determined *in vitro* using rat spleen and brain microsomes as the sources of heme oxygenase-1 and −2, respectively, as well as recombinant, human heme oxygenase-2.

**Results:**

Menadione analogs with bulky aliphatic groups at position-3, namely vitamins K_1_ and K_2,_ were not able to activate HO-2. In contrast, several compounds with similar bulky but less lipophilic moieties at position-2 (and −3) were able to activate HO-2 many fold; these compounds included polar, rigid, furan-containing naphthoquinones, furan-benzoxazine naphthoquinones, 2-(aminophenylphenyl)-3-piperidin-1-yl naphthoquinones. To explore the idea that redox properties might be involved in menadione efficacy, we tested analogs such as 1,4-dimethoxy-2-methylnaphthalene, pentafluoromenadione, monohalogenated naphthoquinones, α-tetralone and 1,4-naphthoquinone. All of these compounds were inactive except for 1,4-naphthoquinone. Menadione activated full-length recombinant human heme oxygenase-2 (FL-hHO-2) as effectively as rat brain enzyme, but it did not activate rat spleen heme oxygenase.

**Conclusions:**

These observations are consistent with the idea that naphthoquinones such as menadione bind to a receptor in HO-2 and activate the enzyme through a mechanism that may involve redox properties.

## Background

Drug targets or drug receptors include a broad array of cellular entities that range from receptors for intercellular transmitters to ion channels to genetic macromolecules to enzymes. Examples of the latter include xanthine oxidase, aldehyde dehydrogenase, angiotensin converting enzyme and acetylcholinesterase for which allopurinol, disulfuram, enalaprilate and organophosphates, respectively, act as ligands. In their 2010 review, Zorn and Wells [1] highlight the observation that inhibition of enzyme function is the usual *modus operandi* of drug discovery programs that target specific enzymes; accordingly in all of the examples given above the ligands inhibited the enzymes. The pursuit of small molecules that activate enzymes is much less common as is the number of therapeutic agents that increase the activity of enzyme molecules.

In the realm of heme oxygenases (HO), much of the progress made toward understanding their functional roles has exploited animals that are genetically deficient in either HO-1 or HO-2, treatments that induce HO-1, or drugs that inhibit these enzymes [2,3]. Through utilization of these tools, we now appreciate that heme oxygenases and their products are involved in an interesting array of cellular activities. For example, considerable evidence revealed that HO-1 affords tissue protection in the vasculature due to the antioxidant, anti-inflammatory and anti-apoptotic properties of its products (see review by Araujo et al. [4]). Similarly, there is substantial evidence that HO-1 protects neurons against oxidative stress [5]. The first generation of HO inhibitors comprises the metalloporphyrins, such as zinc protoporphyrin (ZnPP) and tin protoporphyrin (SnPP), which are powerful inhibitors of both HO-1 and HO-2 [6]. With respect to the cardiovascular system, Araujo et al. [4] have reviewed the evidence showing that HO-1 is protective against vascular inflammation, and cite examples such as the worsening of ischemia reperfusion injury in the presence of ZnPP [7]. Our laboratory has subsequently created a series of azole-based HO inhibitors, many of which are selective for HO-1 [8,9] and more recently a series of compounds that are selective for HO-2 [10]. As was the case for research addressing enzymes as drug targets in general, virtually all of the literature on small molecules that affect heme oxygenases was devoted to inhibitors of these enzymes. An exception to this was our recent report on the *in vitro* activation of HO-2 by menadione [11]. Menadione activated both the rat, native HO-2 and recombinant human HO-2 seven to thirty-fold, and did not activate HO-1. The mechanism of activation of HO-2 was through an increase in maximum velocity and not by a change in affinity for the substrate.

In this communication, we present some structure–activity relationships of menadione and its analogs as activators of HO-2, and test the hypothesis that redox properties of these compounds participate in their activation of HO-2.

## Methods

Native HO-1 and HO-2 were prepared as microsomes from rat spleen and brain as described previously by Vukomanovic et al. [11]. All animals used were cared for in accordance with the principles and guidelines of the Canadian Council on Animal Care and the experimental protocols were approved by the Queen’s University Animal Care Committee. A full-length cDNA clone of full-length human HO-2 (FL-hHO-2) in pOTB7 was obtained from Open Biosystems. Following PCR amplification to engineer *Nde*I and *EcoR*I sites at the 5’ and 3’ ends, respectively, the coding region was subcloned into the pET28a vector using these restriction sites. The resultant recombinant protein contained an N-terminal histidine tag to allow purification by metal chelation using a protocol modified from previous publications [12,13]. Briefly, BL21 (DE3) cells, transformed with the FL-hHO-2/pET28a plasmid, were grown in LB supplemented with 30 μg/mL of kanamycin at 37°C until an OD_600_ of ~ 0.8, at which point protein expression was induced with 1 mM isopropyl β-d-1-thiogalactopyranoside. Cells were grown for a further 3–4 h before harvesting and cell pellets were stored at −80°C until ready to purify. Subsequently, thawed pellets were resuspended in lysis buffer [50 mM NaH_2_PO_4_ (pH 8.0), 300 mM NaCl, lysozyme (0.5 mg/mL; BioShop), DNaseI (5 units/mL; Fermentas), EDTA-free protease inhibitor cocktail (1 tablet/50 mL; Roche)], incubated on ice (30 min) and lysed by sonication. Following centrifugation at 27,000 × g (15,000 rpm in a Beckman JA 25.5 rotor), the resultant pellets were solubilized in lysis buffer containing 1% *n*-Dodecyl β-d-Maltopyranoside (Affymetrix) overnight at 4°C. Solubilized protein was collected from the supernatant following centrifugation at 31,000 × g (16,000 rpm in a Beckman JA 25.5 rotor) and combined with the previously clarified lysate for purification by nickel affinity chromatography using Ni-NTA agarose resin. The combined supernatants were allowed to bind to the resin by rocking for 1 h at 4°C. After collecting the flowthrough, unbound protein was removed by a step-wise imidazole gradient (0–50 mM); protein was eluted by an addition step-wise gradient from 100–500 mM imidazole. Protein-containing fractions were identified by 12% SDS-PAGE and positive fractions (200–500 mM) dialyzed overnight against 20 mM potassium phosphate (pH 7.4), 0.1 mM EDTA, 10% glycerol. Following protein quantification (Bio-Rad), heme was conjugated by slow addition of hemin solution to a final molar ratio of 2:1 as described previously by Rahman et al. [13]. Further purification of the heme-bound enzyme and removal of excess heme was performed by size-exclusion chromatography over an S200 column. Protein concentration was determined by absorbance (ϵ_405_ = 171.4 ± 1.2 mM^-1^ cm^-1^) [13]. Purity was assessed by measurement of the Rz ratio (A_405_/A_280_ > 2.1) and by SDS-PAGE analysis.

HO-1 and HO-2 activities were determined as described by Vukomanovic et al. [11]. In brief, a reaction mixture (150 μL) containing 100 mM phosphate buffer (pH 7.4), 50 μM methemalbumin and 0.5 mg mL^-1^ brain or 1 mg mL^-1^ spleen protein was pre-incubated with an HO activator, concentration as described below, for 10 min at 37°C. Methemalbumin was prepared by dissolving hemin in 10% (w/v) ethanolamine in water, and mixing it with a bovine serum albumin that had been dissolved in water [14,15]. The reaction was initiated by adding 1 mM NADPH and was continued for 15 min at 37°C. The reaction was stopped by instantly freezing the reaction mixture on dry ice, and the generated CO was determined by gas chromatography. For experiments involving FL-hHO-2, the concentration of the enzyme was 0.7 μM and the concentration of purified human NADPH-P450 reductase (recombinant, Becton Dickinson Canada Inc., Toronto, ON, Canada) was 0.01 μM.

### Source of drugs

Drugs and compounds were obtained from the following sources: Sigma-Aldrich (Toronto, ON, Canada)- menadione, vitamin K_2_, NADPH, bovine serum albumin, hemin and L-ascorbic acid; Alfa Aesar- (A Johnson Matthey, Ward Hill, MA, USA)- vitamin K_1_; Tokio Chemical Industry America (Portland, OR, USA)- hydroquinone, 1,4-benzoquinone, and 1,4-naphthoquinone (1,4-naphthalenedione); Acros Organics (Fair Lawn, NJ, USA)- alpha-tetralone, 1,4-cyclohexanedione; Ryan Scientific (Mt. Pleasant, SC, USA)- pentafluoromenadione; Otava Ltd. (Toronto, ON, Canada)- furan-containing naphthoquinones, furan-benzoxazine containing naphthoquinones, 2-aminophenyl naphthoquinones and 3-(piperidin-1-yl) naphtoquionones. 2-Phenylnaphthoquinones were obtained from Snieckus Innovations (Kingston, ON, Canada). 1,4-Dimethoxy-2-methylnaphthalene (methoxy analog of menadione) was a gift from Dr. D. N. Criddle, University of Liverpool, UK.

## Results

**Figure 1:**
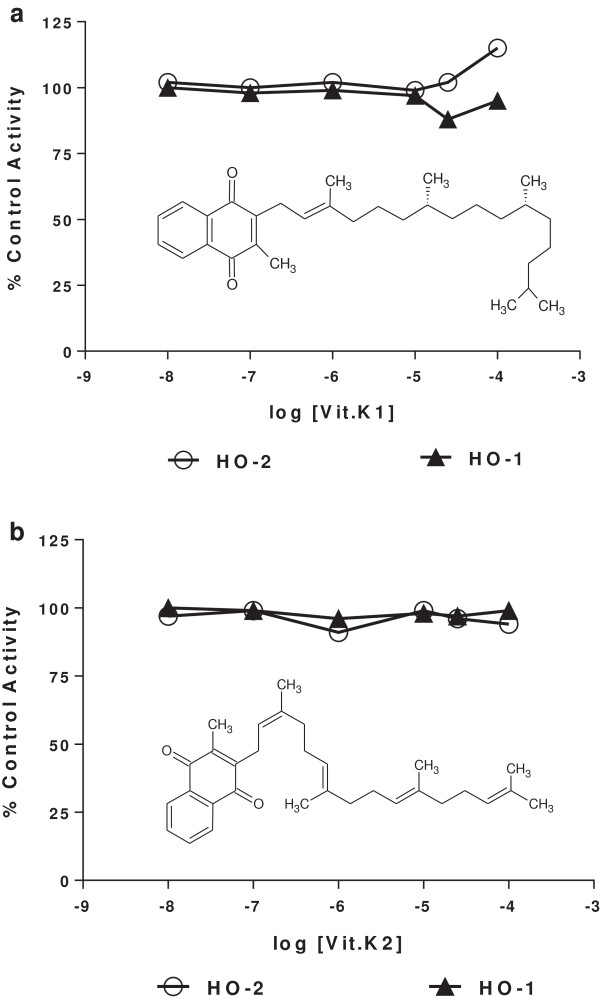
**Lack of effect of vitamins K**_**1 **_**and K**_**2 **_**on HO-1 and HO-2 activity *in vitro.*** HO-1 and HO-2 activity were measured as described in Methods using spleen (solid triangles) and brain (open circles) microsomes, respectively. The abscissa shows drug concentration and ordinate shows HO-2 activity as a percent of control in the absence of added drug. Panel **a**, Vitamin K_1_ and panel **b**, Vitamin K_2_. The structures of vitamins K_1_ and K_2_ in the panels to illustrate their differences from menadione.

**Figure 2:**
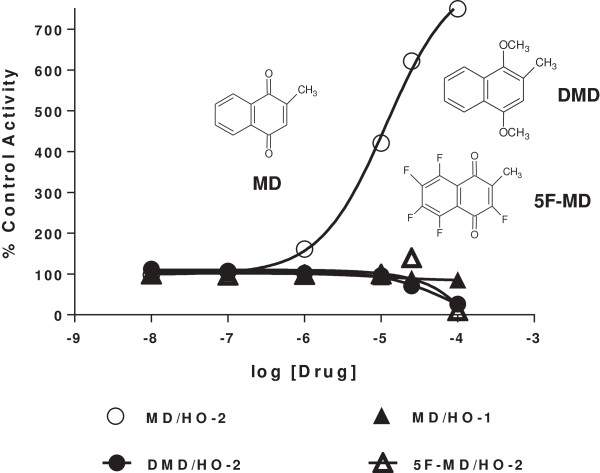
**Activation of rat brain HO-2 by menadione, and lack of activation by dimethoxymenadione (DMD) and pentafluromenadione (5 F-MD).** HO-1 and HO-2 activity were measured as described in Methods using spleen and brain microsomes, respectively. The abscissa shows drug concentration and the ordinate shows HO activity as a percent of control in the absence of added drug. Open circles show menadione activation of HO-2, and solid triangles show lack of activation of rat spleen HO-1. Solid circles show lack of activation of HO-2 by dimethoxy analog of menadione. Open triangles show lack of activation of HO-2 by the fluoro analog of menadione.

An important biological property of menadione is its activity as a K vitamin; thus, we were interested to know if vitamin K_1_ or vitamin K_2_ also activated HO-2. In contrast to menadione, neither vitamin K_1_ nor vitamin K_2_ was able to increase the enzymatic activity of either HO-1 or HO-2 as shown in Figure 1. Thus, the HO-1 and HO-2 activity curves for vitamin K_1_ (panel a) were flat from 10 nM to10 μM with a small upward trend at 100 μM for brain microsomal HO-2, and the curves for vitamin K_2_ (panel b) were flat throughout the full concentration range. In comparison, menadione activated HO-2 about 7-fold (Figure 2). Structurally menadione differs from vitamins K_1_ and K_2_ in that it possesses a methyl moiety at the 2-position whereas vitamins K_1_ and K_2_ each possess an additional, adjacent long unsaturated aliphatic side chain (Figure 1). Because this side chain could possibly interfere with binding of vitamin K_1_ or K_2_ to HO-2 through steric hindrance, we decided to explore the activity of other menadione analogs that possessed large non-aliphatic substituents at positions-2 and −3.

For these experiments, we tested menadione analogs that possessed various substituents at the 2- and 3-positions including- naphthoquinones containing furan-carboxylic acid substituents (Table 1), naphthoquinones containing furan-benzoxazine (Table 2), naphthoquinones containing aminophenyl and piperidinyl moieties (Table 3), naphthoquinones containing 2-phenyl (Table 4) and other naphthoquinones containing various substituents at positions-2 and −3 (Table 5). Tables 1, 2, 3 and 4 show activation of rat brain HO-2 by menadione analogs possessing a wide variety of substituents at the 2- and 3-position. For example, compound 71830417 (a furan-containing naphthoquinone) activated HO-2 to 693% of control, SI-859 (a 2-phenylnaphthoquinone) activated HO-2 to 691%, and 7010100237 (a 2-(aminophenyl)-3-piperidin-1-yl)naphthoquinone) activated HO-2 to 249% of control. Clearly, a variety of moieties could be present at the 2- and 3-positions of naphthoquinone, with maintenance of the ability to activate HO-2. Thus, unlike vitamins K_1_ and K_2_, the mere presence of a large substituent at the 2- and 3-positions in these compounds did not render them unable to activate HO-2.

**Table 1:** HO-2 activation by naphthoquinones containing furan-carboxylic acid substituents

**Structure**	**HO-2**	**HO-1**	**Structure**	**HO-2**	**HO-1**
	**% Act.**	**% Act.**		**% Act.**	**% Act.**
7110952633	**276**	**96**	7118340386	**153**	**93**
7118340392	**288**	**85**	7118340387	**320**	**91**
7118340396	**326**	**78**	7118340385	**578**	**72**
7118340391	**368**	**80**	7118340381	**508**	**89**
7118340395	**457**	**66**	7118340384	**540**	**100**
7118340416	**548**	**79**	7118340380	**533**	**86**
7118340415	**566**	**79**	7118340379	**560**	**85**
7116980337	**586**	**84**	718340374	**602**	**91**
7118340417	**693**	**71**			

Rat HO-1 and HO-2 activities were determined as described in Methods. Test compounds were used at a concentration of 100 μM. Activity is reported as a percentage of activity in the absence of test compound; control = 100%. The 10-digit numbers are Otava catalog numbers.

**Table 2:** HO-2 activation by naphthoquinones containing furan-benzoxazine moieties

**Structure**	**HO-2**	**HO-1**	**Structure**	**HO-2**	**HO-1**
	**% Act.**	**% Act.**		**% Act.**	**% Act.**
7114420188	**165**	**39**	7114420195	**188**	**26**
7114420189	**165**	**32**	7114420197	**178**	**51**
7114420191	**170**	**29**	7114420193	**173**	**69**
7114420406	**179**	**28**	7114420405	**345**	**85**

Rat HO-1 and HO-2 activities were determined as described in Methods. Test compounds were used at a concentration of 100 μM. Activity is reported as a percentage of activity in the absence of test compound; control = 100%. The 10-digit numbers are Otava catalog numbers.

**Table 3:** HO-2 activation by naphthoquinones containing aminophenyl-piperidinyl substituents

**Structure**	**HO-2**	**HO-1**	**Structure**	**HO-2**	**HO-1**
	**% Act.**	**% Act.**		**% Act.**	**% Act.**
7010100239	**175**	**81**	7010100237	**249**	**82**
7010100238	**192**	**71**	7010100234	**171**	**82**

Rat HO-1 and HO-2 activities were determined as described in Methods. Test compounds were used at a concentration of 100 μM. Activity is reported as a percentage of activity in the absence of test compound; control = 100%. The 10-digit numbers are Otava catalog numbers.

**Table 4:** HO-2 activation by naphthoquinones containing 2-phenyl moieties

**Structure**	**HO-2**	**HO-1**	**Structure**	**HO-2**	**HO-1**
	**% Act.**	**% Act.**		**% Act.**	**% Act.**
SI-859	**691**	**81**	SI-2522	**484**	**79**
SI-108	**643**	**95**	SI-556	**455**	**64**
SI-1606	**596**	**84**	SI-1186	**350**	**91**
SI-102	**560**	**102**			

HO-1 and HO-2 activities were determined as described in Methods. Test compounds were used at a concentration of 100 μM. Activity is reported as a percentage of activity in the absence of test compound; control = 100%. MOMO is methoxymethyl ether. SI = Sniekus Innovations.

**Table 5:** Lesser HO-2 activation by naphthoquinones containing various additional moieties

**Structure**	**HO-2**	**HO-1**	**Structure**	**HO-2**	**HO-1**
	**% Act.**	**% Act.**		**% Act.**	**% Act.**
7116411293	**107**	**99**	7010100229	**110**	**90**
7116411335	**108**	**100**	70101100227	**95**	**76**
7116411344	**105**	**97**	7010100231	**127**	**73**
7010100236	**113**	**93**	7010100235	**120**	**90**
7010100240	**102**	**95**	7210710091	**92**	**17**
7010100225	**119**	**74**			

HO-1 and HO-2 activities were determined as described in Methods. Test compounds were used at a concentration of 100 μM. Activity is reported as a percentage of activity in the absence of test compound; control = 100%. The 10-digit numbers are Otava catalog numbers.

### Mechanism of action

According to classical receptor theory, an agonist drug such as menadione possesses affinity for its receptor on HO-2 and efficacy once it has bound. The inactivity of vitamin K_1_ might be explained by an inability to bind to HO-2. To explore this, we tested the effects of menadione in the presence of vitamin K_1_. This experiment was done in two ways- by adding vitamin K_1_ (10 – 100 μM) to the reaction mixture before menadione (25 μM) or after menadione. Both iterations yielded no effect on menadione activation of HO-2; vitamin K_1_ did not alter the menadione-induced activation of HO-2 (data not shown).

An obvious characteristic of menadione and its analogs studied herein is their naphthoquinone core, which is known to participate in redox reactions [16]. Consequently, we obtained four menadione analogs in which redox activity was compromised to determine if there was a direct correlation between redox properties and activation of HO-2. One such analog was 1,4-dimethoxy-2-methylnaphthalene wherein the keto oxygens of menadione are replaced by methoxy groups; this analog did not activate HO-2 as shown in Figure 2. Another analog used was pentafluoromenadione, which would be compromised in redox reactions due to the strong electron-withdrawing action of the five fluorine atoms; pentafluoromenadione was without HO-2 stimulating activity (Figure 2). Table 6 shows the lack of activation of HO-2 by 2-bromo-5-hydroxynaphthoquinone and 2-chloro-3-hydroxynaphthoquinone. To explore further the relationship between redox properties and activation of HO-2, we tested two series of benzoquinone- and naphthoquinone-like molecules as shown in Table 7. Thus, within the triad of 1,4-cyclohexanedione, hydroquinone and 1,4-benzoquinone, there was a major difference in effect on HO-2 activity. Those compounds that were poor candidates for redox activity, namely 1,4-cyclohexanedione and hydroquinone, were essentially inactive toward both HO-1 and HO-2. Meanwhile 1,4-benzoquinone did not activate HO-2, but was a strong inhibitor of both HO-1 and HO-2. Of the α-tetralone and 1,4-naphthoquinone pair, α-tetralone was inactive toward HO-1 and HO-2. 1,4-Naphthoquinone had little effect on HO-1, but activated HO-2 five-fold.

**Table 6:** Monohalogenated, monohydroxynaphthoquinones are inactive as HO activators

**Structure**	**HO-2**	**HO-1**	**Structure**	**HO-2**	**HO-1**
	**% Act.**	**% Act.**		**% Act.**	**% Act.**
7119850429	**108**	**106**	SI-441	**97**	**49**

HO-1 and HO-2 activities were determined as described in Methods. Test compounds were used at a concentration of 100 μM. Activity is reported as a percentage of activity in the absence of test compound; control = 100%. The 10-digit number is an Otava catalog number; SI = Sniekus Innovations.

**Table 7:** HO activity in the presence of benzoquinone-like and naphthoquinone-like compounds

**Structure**	**% Act. HO-2 @ 100 μm**	**% Act. HO-1 @ 100 μm**	**Structure**	**% Act. HO-2 @ 100 μm**	**% Act. HO-1 @ 100 μm**
1,4-cyclohexandione	**105**	**103**	α-tetralone	**96**	**98**
1,4-benzoquinone	**2**	**-5**	1,4-naphthoquinone	**503**	**71**
hydroquinone	**108**	**102**			

HO-1 and HO-2 activities were determined as described in Methods. Test compounds were used at a concentration of 100 μM. Activity is reported as a percentage of activity in the absence of test compound; control = 100%.

### Menadione activation of recombinant human HO-2

**Figure 3:**
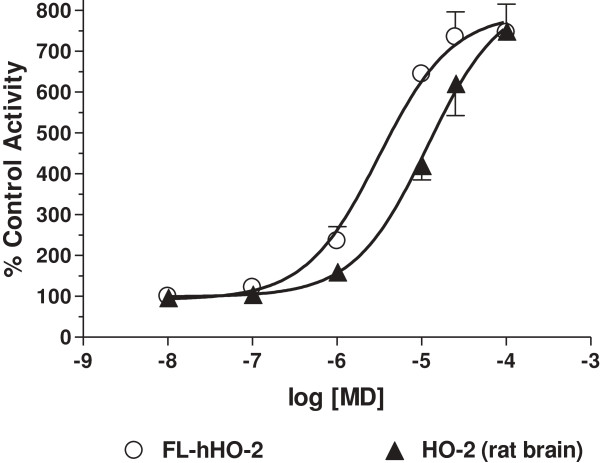
**Menadione activated full-length human, recombinant HO-2 similar to rat native HO-2.** HO-2 activity was measured as described in Methods using recombinant hHO-2 (open circles) and rat brain microsomes (closed triangles) as the source of HO-2. The abscissa shows drug concentration and ordinate shows HO-2 activity as a percent of control in the absence of added drug (mean ± SD, n = 4).

Our drug design work has relied heavily on the rat, native HOs, in part because the native enzymes were crucial in allowing us to create selective inhibitors of HO-1 [11]. Nevertheless, the ultimate application of knowledge to human health requires one to explore the novel effects of drugs in the human context. Furthermore, working with a purified recombinant protein sample provides insights at the molecular level that is often challenging when using a mixture. Accordingly, we tested the effects of menadione on recombinant human HO-2. Figure 3 shows that the FL-hHO-2, was activated ~7-fold by menadione, which compares well with menadione-activation of the rat, native HO-2 (also ~ 7-fold).

## Discussion

The goals of this work were to explore the structure-activity relationship of menadione activation of HO-2 and to test the hypothesis that redox properties of menadione and its analogs participate in their activation of heme oxygenase-2. The main observations made in the present study were- (a) HO-2 was not activated by vitamins K_1_ and K_2_ but was activated by menadione (vitamin K_3_), (b) HO-2 was activated by furan carboxylic acid-containing naphthoquinones, furan-benzoxazine-containing naphthoquinones, 2-phenylnaphthoquinones, and 2-(aminophenyl)-3-(piperidin-1-yl)naphthoquinones, (c) the dimethoxy, pentafluoro and monohalogenated analogs of menadione did not activate HO-2, (d) 1,4-naphthoquinone strongly activated HO-2, and (e) full-length human recombinant HO-2 was activated to a similar extent as rat, native HO-2.

The absence of HO-2 activation by vitamins K_1_ and K_2_ indicates that the receptor responsible for HO-2 activation by menadione differs from the receptor for vitamin K activity. As stated above, this lack of HO-2 activation by vitamins K_1_ and K_2_ could have been due to their bulky aliphatic side chains sterically hindering binding to the enzyme or lack of inducing an appropriate activating conformational change of HO-2. The observation that addition of both menadione and vitamin K_1_ to the *in vitro* reaction mixture resulted in activation of HO-2 similar to that of menadione alone is consistent with the notion that vitamin K_1_ does not bind to the same site on HO-2 as does menadione. Had vitamin K_1_ bound to the menadione binding site, it would have antagonized the activating effect of menadione. Nevertheless, we acknowledge the fact that binding experiments with a radiolabelled ligand were not conducted.

This inability of vitamin K_1_ to bind to the menadione binding site on HO-2 does not appear to be due to steric hindrance alone because the side chains attached to the compounds listed in Tables 1, 2, 3, 4 are similar in size to the aliphatic side chains of vitamin K_1._ While these compounds are similar to vitamins K_1_ and K_2_ with respect to the presence of a significantly large moiety at carbon-2, they differ substantially in the nature of the substituent; these compounds contain hetero-atoms that would increase polarity. If there are polar sites on HO-2 in the region of the menadione binding site, they might be compatible with heteroatom-containing side chains but incompatible with non-polar moieties such as the aliphatic side chain of vitamin K_1_.

The hypothesis that redox properties of menadione and its analogs participate in their activation of HO-2 arose from the well-known redox properties of the quinone nucleus [16]. Our observation that the dimethoxy analog of menadione (1,4-dimethoxy-2-methylnaphthalene) was unable to activate HO-2 is consistent with the hypothesis. In the case of 1,4-dimethoxy-2-methylnaphthalene, the absence of the quinone nucleus would block the ability of this molecule to participate in redox reactions [17]. In the case of pentafluoromenadione, the presence of the five fluorine atoms causes a large change in the behaviour of electrons (negative inductive effect) of the naphthoquinone nucleus; accordingly, the reduction potential of pentafluoromenadione would be in the order of 400 mV compared with −203 mV for menadione [18]. This reduction potential for the pentafluorinated analog of menadione would obviate redox function. Further support for this line of reasoning may be found in the lack of HO-2 activation by the two monohalogenated, monohydroxy naphthoquinone analogs shown in Table 6, which were found not to activate either HO-2 or HO-1. The hydroxyl groups on the latter two molecules could also contribute to this loss of HO-2 activation through resonance-based decreases in redox reactions. The HO-2 data obtained with these four compounds are consistent with the hypothesis as articulated above.

Another approach taken was to test the effects of five commercially available benzoquinone-like and naphthoquinone-like compounds on HO activation as shown in Table 7. Based on their chemical structures, we considered the hypothesis to predict that α-tetralone would be a poor activator of HO-2 because it would not be active with respect to redox activity. Similarly, 1,4-cyclohexanedione and hydroquinone were predicted to be inactive as HO-2 activators. The hypothesis predicted that 1,4-naphthoquinone would be fully active as an HO-2 activator because it possesses the complete quinone nucleus that is essential for redox reactions. While 1,4-benzoquincbone shares a quinone nucleus with 1,4-naphthoquinone, it was anticipated to be inactive because it has been observed not to participate in redox reactions but rather it arylates protein thiols [19]. Thus, the data obtained from these five compounds are consistent with the hypothesis articulated above. On balance, it appears that HO-2 activating naphthoquinones require both affinity for a selective receptor and redox activity.

The observations that these naphthoquinones activated HO-2 but did not activate HO-1 are interesting from both mechanistic and application perspectives; certainly they should be useful for elucidating the roles of HO-1 and HO-2 in various physiological and metabolic functions. This observation also indicates that these HO-2 activators bind to a site on HO-2 other than the catalytic site because of the high conservation of amino acid sequence between HO-1 and HO-2 therein. We have excluded cytochrome P450 reductase as the activation site by observing that hHO-2 (1–288) activation occurred (>200%) in the absence of NADPH and cytochrome P450 reductase whereby the source of reducing capacity was ascorbic acid (data not shown). In the present experiments, the source of reducing equivalents would include NADPH. In the case of enzyme inhibitors the locus of drug binding is often at or near the active site, as would be the case for competitive inhibitors. In the case of enzyme activators, the binding site should not be the same as that of the natural substrate (i.e. active site), because binding of a non-substrate molecule there must result in competitive inhibition. It follows that an enzyme activator must bind beyond the active site, but still influence the reaction in a positive manner.

## Conclusions

The activation of HO-2 by menadione and similar agonists requires affinity for a receptor site outside of the enzyme core (which it shares with HO-1) and probably also the ability to participate in redox reactions.

## Abbreviations

HO: Heme oxygenase; HO-1: Heme oxygenase-1; HO-2: Heme oxygenase-2; hHO-2: Recombinant human heme oxygenase-2; ZnPP: Zinc protoporphyrin; SnPP: Tin protoporphyrin.

## Competing interests

None declared for Vukomanovic, Rahman, Brien and Jia.

Nakatsu and Szarek hold a patent on selective heme oxygenase-1 inhibitors.

Bilokin and Golub are affiliated with Otava.

## Authors’ contributions

Participated in research design: N, V. Conducted experiments: V, R. Contributed new reagents or analytic tools: B, G. Performed data analysis: N, V. Wrote or contributed to the writing of the manuscript: N, V, B, J, S. All authors read and approved the final manuscript.
